# Concentrations of estradiol, progesterone and testosterone in sefrum and cerebrospinal fluid of patients with aneurysmal subarachnoid hemorrhage correlate weakly with transcranial Doppler flow velocities

**DOI:** 10.1186/s12868-021-00634-3

**Published:** 2021-04-23

**Authors:** Jan Martin, Eva Plank, Bernhard Ulm, Jens Gempt, Maria Wostrack, Bettina Jungwirth, Simone M. Kagerbauer

**Affiliations:** 1 Department of Anesthesiology and Intensive Care Medicine, School of Medicine, Klinikum Rechts Der Isar, Technical University of Munich, Ismaninger Str. 22, 81675 Munich, Germany; 2 Department of Neurosurgery, School of Medicine, Klinikum Rechts Der Isar, Technical University of Munich, Ismaninger Str. 22, 81675 Munich, Germany; 3 Department of Anesthesiology and Intensive Care Medicine, Ulm University, Albert-Einstein-Allee 23, 89081 Ulm, Germany

**Keywords:** Aneurysmal subarachnoid hemorrhage, Vasospasm, Estradiol, Progesterone, Testosterone, Cerebrospinal fluid, Blood, Human

## Abstract

**Background:**

The implication of the steroids estradiol, progesterone and testosterone in cerebral vasospasm after aneurysmal subarachnoid hemorrhage (aSAH) has not been comprehensively assessed. In rodents, studies suggested beneficial effects of steroids on cerebral vasospasm after experimental SAH. Studies in humans are warranted, however, a general dilemma of human studies on neuroactive substances is that the brain is not directly accessible and that concentrations in the periphery may not adequately parallel concentrations in the central compartments. In the present study, concentrations of estradiol, progesterone and testosterone in serum and cerebrospinal fluid (CSF) of patients with aSAH were determined. Blood flow velocities in cerebral arteries were measured by transcranial Doppler sonography (TCD). The aim of this study was to evaluate the correlations between the cerebral blood flow velocities and levels of estradiol, progesterone and testosterone in CSF and serum.

**Results:**

Samples of serum and CSF of 42 patients with aSAH were collected concomitantly daily or every other day via the arterial line and the external ventricular drainage for two weeks after the hemorrhage. Blood flow velocities in the cerebral arteries were determined by TCD. Total estradiol, progesterone and testosterone concentrations were measured by electro-chemiluminescence immunoassay. The strength of correlation was assessed by Spearman’s rank correlation coefficient. The correlation analysis revealed very weak correlations between cerebral blood flow velocities and concentrations of estradiol, progesterone and testosterone levels in both compartments with correlation coefficients below 0.2.

**Conclusions:**

In humans with aSAH, merely very weak correlations between flow velocities in cerebral arteries and concentrations of estradiol, progesterone and testosterone in serum and CSF were demonstrated. These results suggest a limited influence of the respective steroids on cerebral vascular tone although vasodilatory effects were described in rodent studies. Thus, the implication of steroids in processes of neurological deterioration warrants further clarification.

## Background

Aneurysmal subarachnoid hemorrhage (aSAH) refers to the spontaneous rupture of an intracranial aneurysm. Despite improvements in microsurgery, interventional neuroradiology and neurocritical care the disease continues to be associated with a high risk of morbidity and mortality. The issue of delayed neurological deterioration after aSAH has long been addressed and remains subjected to studies in order to shed light on a complex system of processes due to cerebral vasospasm and due to processes that seem to be unrelated to cerebral vasospasm [1, 2].

In the development of neurological deterioration due to cerebral vasospasm after aSAH an endothelial damage or inflammatory causes are commonly discussed [2, 3]. Steroids appear involved in these processes but it is still largely unsettled to what extent the steroids estradiol, progesterone and testosterone have an influence on the development or degree of cerebral vasospasm. In rodents, several studies assessed the potentially beneficial effects of these steroids on cerebral vasospasm after experimental subarachnoid hemorrhage (SAH) [2, 4, 5]. A further knowledge of the implication of estradiol, progesterone and testosterone in differing compartments, such as blood, cerebrospinal fluid (CSF) and brain parenchyma in influencing cerebral vasospasm may help to understand factors underlying its pathogenesis or unveil therapeutic options. Studies in humans are warranted, however, a particular problem of studies in humans on centrally acting substances is that the brain is not easily accessible and that concentrations in periphery, e.g. blood, may not adequately parallel concentrations in brain parenchyma. Presuming that steroid concentrations in the CSF may reflect steroid activity in the brain, this problem can be approached but basically remains. Also, human CSF is not easily obtained and normally invasive techniques such as lumbar puncture or ventricular drainage are needed whereby the latter is often performed in aSAH patients as a therapeutic measure.

In the present study, concentrations of estradiol, progesterone and testosterone in CSF and blood of patients with aSAH were determined. Blood flow velocities in cerebral arteries were measured by transcranial Doppler sonography. The aim of this study was to assess neuroprotective vasodilatory influences attributed to the steroids estradiol, progesterone and testosterone by evaluating the correlations between the blood flow velocities in the cerebral arteries and levels of the respective steroids in CSF and serum.

## Methods

The study was approved by the ethics committee of the medical faculty of the Technical University Munich (project number 2410/09). We prospectively studied 42 patients (mean age 57 years, range 20 to 80 years) with aneurysmal SAH (Table 1). Written informed consent was provided by all patients or their legal guardians respectively. The patient’s clinical condition after the onset of the disease was assessed by the grading scale of Hunt &Hess [6]. The blood flow velocities in the anterior, middle and posterior cerebral arteries were measured by transcranial Doppler sonography (TCD). Cerebral infarction was diagnosed by computer tomography. Patient outcome was assessed on the Glasgow Outcome Scale [7] at three months after the ictus.

**Table 1:** Patient characteristics

	Age	Sex	HH	Aneurysm location	Treatment	TCD range	CI	GOS
1	45–54	F	3	ACA	Coiling	[100, 300]	No	5
2	55–64	F	5	ACoA	Coiling	[120, 220]	Yes	2
3	45–54	F	3	PCoA	Coiling	[120, 220]	No	5
4	75–84	F	3	ICA	Clipping	[120, 200]	No	5
5	55–64	M	5	MCA	Clipping	[140, 300]	Yes	3
6	55–64	F	3	PCoA	Coiling	[120, 220]	No	5
7	65–74	F	5	PCoA	Coiling	[140, 200]	Yes	1
8	45–54	F	4	VA	Coiling	[100, 220]	No	5
9	45–54	M	3	PCoA	Coiling	[130, 310]	No	5
10	45–54	F	2	ACoA	Coiling	[80, 250]	No	5
11	25–34	F	1	PCoA	Coiling	[75, 320]	No	5
12	35–44	F	2	ACA	Clipping	[120, 300]	Yes	3
13	35–44	M	2	PCoA	Clipping	[200, 340]	Yes	3
14	45–54	M	3	MCA	Clipping	[120, 270]	No	3
15	65–74	F	2	PICA	Clipping	[180, 300]	No	3
16	55–64	F	2	PCoA	Clipping	[160, 220]	No	5
17	45–54	M	5	MCA	Clipping	[220, 350]	Yes	3
18	65–74	M	2	ACoA	Coiling	[130, 200]	Yes	5
19	65–74	F	3	ACA	Clipping	[100, 330]	Yes	3
20	45–54	F	4	ACoA	Clipping	[120, 220]	Yes	4
21	65–74	F	3	ACA	Clipping	[120, 220]	Yes	4
22	55–64	F	5	MCA	Clipping	[130, 240]	No	3
23	75–84	F	2	ACoA	Coiling	[130, 150]	No	4
24	25–34	F	2	MCA	Clipping	[70, 150]	No	5
25	25–34	F	3	ICA	Coiling	[100, 180]	No	5
26	65–74	F	3	MCA	Coiling	[70, 170]	No	2
27	65–74	F	3	ICA	Coiling	[170, 200]	No	3
28	55–64	F	5	ACoA	Coiling	[100, 250]	No	4
29	65–74	F	4	PCoA	Clipping	[100, 190]	No	3
30	45–54	F	2	BA	Coiling	[120, 250]	No	5
31	55–64	F	5	MCA	Clipping	[130, 250]	Yes	2
32	15–24	F	2	MCA	Clipping	[160, 350]	No	5
33	45–54	M	5	ACA	Clipping	[90, 300]	Yes	4
34	65–74	F	3	ACoA	Clipping	[110, 130]	No	5
35	45–54	F	4	ACA	Clipping	[110, 160]	No	1
36	65–74	F	4	ACoA	Clipping	[80, 170]	No	4
37	75–84	F	5	MCA	Clipping	[140, 170]	No	1
38	45–54	F	2	PCoA	Coiling	[220, 280]	No	5
39	75–84	F	5	BA	Coiling	[200, 320]	Yes	1
40	55–64	F	3	PCoA	Coiling	[110, 220]	No	5
41	45–54	F	3	MCA	Clipping	[130, 180]	No	4
42	65–74	F	4	MCA	Clipping	[220, 270]	Yes	1

Patient characteristics of 42 patients with aneurysmal subarachnoid hemorrhage. Patient age is given an age range. The TCD data refer to the range of the maximum systolic flow velocities (cm/sec) measured by transcranial Doppler sonography of cerebral arteries (ACA, MCA, PCA) during fourteen days after the index bleeding. The GOS value refers to the score at three months after hospital discharge (GOS 1, death; GOS 2, vegetative state; GOS 3, severe disability; GOS 4, moderate disability; GOS 5, good recovery)

*ACA* anterior cerebral artery, *ACoA* anterior communicating artery, *BA* basilar artery, *CI* cerebral infarction, *CSF* cerebrospinal fluid, *F* female, *GOS* Glasgow Outcome Score, *HH* Hunt and Hess Scale, *ICA* internal carotid artery, *M* male, *MCA* middle cerebral artery, *PCoA* posterior communicating artery, *PICA* posterior inferior cerebellar artery, *TCD* transcranial Doppler, *VA* vertebral artery

Samples of CSF and serum were collected concomitantly daily or every other day in the morning via the arterial line and the external ventricular drainage during the first two weeks after the hemorrhage thereby generally covering the period of maximal cerebral vasospasm [1, 2]. Total estradiol, progesterone and testosterone concentrations in CSF and serum were measured on a Roche Mannheim Cobas e 411 immunoassay analyzer using an electro-chemiluminescence immunoassay (detection limit of 5 pg/ml for estradiol, detection limit of 0.03 ng/ml for progesterone, detection limit of 0.025 ng/ml for testosterone). The TCD measurements were performed daily or every other day in the morning at the time of sample collection.

Strength of bivariate monotonous correlation of quantitative data was assessed by Spearman’s rank correlation coefficient. Correlations were analyzed between the maximum systolic blood flow velocity determined in a cerebral artery and the concentrations of estradiol, progesterone and testosterone in serum und CSF respectively per measurement day. The calculation of each correlation coefficient was based on 281–286 observations according to the steroid and the compartment investigated. An absolute value of the correlation coefficient of 0.00–0.19 indicates a very weak correlation, 0.20–0.39 a weak correlation, 0.40–0.59 a moderate correlation, 0.60–0.79 a strong correlation and a value of 0.80–1.0 a very strong correlation.

Group comparisons were performed to evaluate associations between steroid concentrations in the respective compartments and the grading of the Hunt & Hess scale, the grading of the Glasgow Outcome scale and the occurrence of cerebral infarction. The two-group-comparisons of steroid concentrations in serum and CSF and patients with and without cerebral infarction were conducted using the Mann Whitney test. For the multi-group-comparisons (Hunt & Hess scale grades, Glasgow Outcome scale grades) Kruskal–Wallis-testing was used. Hypothesis testing was performed on two-sided 5% significance levels. Statistical analysis was done by using R software 3.5.0 (R Foundation for Statistical Computing, Austria, Vienna).

## Results

The patient characteristics are presented in Table 1. The data on concentrations of estradiol, progesterone and testosterone in serum and CSF are listed in Table 2. The mean concentrations and standard deviations of estradiol, progesterone and testosterone of the overall cohort are demonstrated in Table 3.

**Table 2:** Patient data

	Estradiol (pg/ml)		Progesterone (ng/ml)		Testosterone (ng/ml)	
	In serum	In CSF	In serum	In CSF	In serum	In CSF
1	11.6 ± 4.4	18.8 ± 3.7	0.26 ± 0.152	< 0.03 ± 0	0.14 ± 0.057	0.18 ± 0.045
2	8.4 ± 3.3	16.5 ± 3.0	0.24 ± 0.102	< 0.03 ± 0	0.30 ± 0.144	0.20 ± 0
3	8.4 ± 4.7	22.3 ± 2.6	0.32 ± 0.307	< 0.03 ± 0	0.29 ± 0.105	0.22 ± 0.026
4	18.5 ± 5.7	no data	0.09 ± 0.005	no data	0.12 ± 0.047	no data
5	14.4 ± 6.4	22.8 ± 5.1	0.47 ± 0.323	< 0.03 ± 0	0.57 ± 0.447	0.26 ± 0.129
6	11.1 ± 4.7	22.6 ± 4.4	0.09 ± 0.005	< 0.03 ± 0	0.20 ± 0.070	0.20 ± 0
7	10.0 ± 7.2	20.3 ± 9.6	0.19 ± 0.268	< 0.03 ± 0	0.15 ± 0.090	0.20 ± 0
8	5.7 ± 2.2	16.5 ± 6.0	0.44 ± 0.524	0.038 ± 0.021	0.17 ± 0.072	0.26 ± 0.052
9	7.2 ± 3.6	15.6 ± 4.5	0.36 ± 0.336	< 0.03 ± 0	1.39 ± 0.997	0.27 ± 0.076
10	< 5.0 ± 0	14.0 ± 3.1	0.42 ± 0.147	< 0.03 ± 0	0.15 ± 0.055	0.23 ± 0.052
11	21.1 ± 11.8	13.6 ± 3.2	0.33 ± 0.175	< 0.03 ± 0	0.46 ± 0.400	0.20 ± 0.093
12	33.4 ± 30.4	12.4 ± 2.0	0.11 ± 0.039	< 0.03 ± 0	0.09 ± 0.004	0.14 ± 0.052
13	6.6 ± 2.9	12.1 ± 5.0	0.46 ± 0.251	0.036 ± 0.015	0.46 ± 0.246	0.12 ± 0.044
14	13.3 ± 7.9	13.5 ± 5.0	0.53 ± 0.219	0.033 ± 0.011	0.44 ± 0.119	0.14 ± 0.052
15	15.7 ± 10.0	20.1 ± 7.7	0.52 ± 0.230	0.030 ± 0.004	0.24 ± 0.116	0.18 ± 0.044
16	12.1 ± 3.7	17.7 ± 7.1	0.29 ± 0.186	< 0.03 ± 0	0.27 ± 0.076	0.17 ± 0.049
17	25.2 ± 18.9	18.1 ± 3.4	0.16 ± 0.121	< 0.03 ± 0	0.22 ± 0.135	0.11 ± 0.034
18	5.9 ± 1.6	16.1 ± 3.7	0.17 ± 0.069	< 0.03 ± 0	0.67 ± 0.302	0.09 ± 0.028
19	8.8 ± 3.4	12.0 ± 6.6	0.31 ± 0.361	0.036 ± 0.022	0.21 ± 0.089	0.13 ± 0.048
20	18.1 ± 14.8	14.4 ± 3.8	0.22 ± 0.150	< 0.03 ± 0	0.16 ± 0.143	0.12 ± 0.044
21	9.2 ± 3.5	15.4 ± 3.2	0.37 ± 0.158	0.031 ± 0.007	0.12 ± 0.045	0.13 ± 0.050
22	24.3 ± 26.8	15.2 ± 5.1	0.27 ± 0.164	0.035 ± 0.016	0.25 ± 0.139	0.10 ± 0
23	5.8 ± 1.3	16.5 ± 6.1	0.32 ± 0.181	0.057 ± 0.086	0.09 ± 0.005	0.12 ± 0.042
24	11.0 ± 0	9.9 ± 0	0.80 ± 0	< 0.03 ± 0	0.40 ± 0	0.20 ± 0
25	20.0 ± 10.6	13.9 ± 1.7	0.24 ± 0.127	0.053 ± 0.065	0.14 ± 0.083	0.127 ± 0.05
26	3.8 ± 2.0	10.6 ± 4.0	0.55 ± 0.262	< 0.03 ± 0	0.16 ± 0.074	0.10 ± 0
27	< 5.0 ± 0	12.3 ± 1.5	0.15 ± 0.074	< 0.03 ± 0	0.10 ± 0.037	0.10 ± 0
28	21.0 ± 23.2	15.0 ± 1.6	0.17 ± 0.071	0.031 ± 0.007	0.11 ± 0.036	0.10 ± 0
29	8.4 ± 4.0	15.2 ± 4.7	0.28 ± 0.223	0.031 ± 0.007	0.19 ± 0.094	0.18 ± 0.044
30	9.3 ± 4.6	16.8 ± 4.0	0.12 ± 0.071	< 0.03 ± 0	0.10 ± 0.034	0.12 ± 0.042
31	32.7 ± 5.6	17.3 ± 5.0	0.23 ± 0.144	< 0.03 ± 0	0.14 ± 0.056	0.12 ± 0.044
32	30.0 ± 14.3	16.1 ± 3.1	0.51 ± 0.528	0.086 ± 0.122	0.31 ± 0.176	0.16 ± 0.088
33	7.9 ± 3.1	16.4 ± 1.7	0.20 ± 0.173	< 0.03 ± 0	0.49 ± 0.298	0.11 ± 0.033
34	7.1 ± 2.5	11.3 ± 3.2	0.35 ± 0.131	< 0.03 ± 0	0.17 ± 0.105	0.10 ± 0
35	49.6 ± 41.2	18.8 ± 8.6	0.80 ± 0.424	0.125 ± 0.106	1.30 ± 0.283	0.35 ± 0.212
36	6.0 ± 2.1	11.0 ± 4.4	0.10 ± 0.034	< 0.03 ± 0	0.09 ± 0.003	0.10 ± 0.004
37	11.7 ± 6.2	17.6 ± 4.5	0.58 ± 0.098	0.041 ± 0.029	0.28 ± 0.075	0.20 ± 0.063
38	6.0 ± 1.7	11.1 ± 2.9	0.53 ± 0.125	< 0.03 ± 0	0.17 ± 0.048	0.11 ± 0.032
39	24.6 ± 13.2	24.8 ± 5.3	0.73 ± 0.618	0.047 ± 0.02	0.27 ± 0.174	0.17 ± 0.050
40	10.7 ± 10.9	20.8 ± 2.0	0.57 ± 0.466	0.057 ± 0.055	0.14 ± 0.076	0.12 ± 0.044
41	45.9 ± 58.6	17.3 ± 4.8	0.59 ± 0.679	< 0.03 ± 0	0.29 ± 0.138	0.14 ± 0.052
42	23.1 ± 19.1	22.0 ± 7.1	0.14 ± 0.077	0.037 ± 0.024	0.30 ± 0.152	0.20 ± 0.071

Mean concentrations and standard deviations per patient of estradiol, progesterone and testosterone in serum and CSF

*CSF* cerebrospinal fluid

**Table 3:** Descriptive statistics of the cohort

		Mean (sd)
Estradiol	In serum (pg/ml)	14.5 (16.5)
	In CSF (pg/ml)	16.4 (5.7)
Progesterone	In serum (ng/ml)	0.33 (0.304)
	In CSF (ng/ml)	0.04 (0.031)
Testosterone	In serum (ng/ml)	0.27 (0.304)
	In CSF (ng/ml)	0.15 (0.071)

Mean concentrations and standard deviations of estradiol, progesterone and testosterone of the cohort (n = 42)

*CSF* cerebrospinal fluid, *sd* standard deviation

The Spearman’s rank correlation coefficients between the transcranial Doppler flow velocities and the concentrations of the steroids estradiol, progesterone and testosterone are presented in Table 4. In the overall cohort, the correlation analysis showed merely very weak correlations between cerebral flow velocities and levels of estradiol, progesterone and testosterone in any of the two compartments with correlation coefficients below 0.2 (Table 4). A complementary gender specific correlation analysis revealed no further aspects as the correlation coefficients remained throughout weak for males (< 0.3) and throughout very weak for females (< 0.2).

**Table 4:** Correlation analysis

Estradiol	In serum	Rho = 0.10	p = 0.08
	In CSF	Rho = 0.15	p = 0.01
Progesterone	In serum	Rho = -0.05	p = 0.39
	In CSF	Rho = 0.07	p = 0.25
Testosterone	In serum	Rho = 0.15	p = 0.01
	In CSF	Rho = 0.06	p = 0.34

Spearman rank correlation analysis of concentrations of estradiol, progesterone and testosterone in serum and CSF with transcranial Doppler velocities demonstrated very weak correlations. An absolute value of the correlation coefficient (rho) of 0.00–0.19 indicates a very weak correlation, 0.20–0.39 a weak correlation, 0.40–0.59 a moderate correlation, 0.60–0.79 a strong correlation and a value of 0.80–1.0 a very strong correlation

*CSF* cerebrospinal fluid

The group comparisons between steroid concentrations in the respective compartments and the grading of the Hunt & Hess scale, the grading of the Glasgow Outcome scale and cerebral infarction revealed no significant differences.

## Discussion

In humans with aSAH, possible interrelations between cerebral vasospasm and the steroids estradiol, progesterone and testosterone in peripheral compartments, e.g. blood, or in central compartments, e.g. CSF, have not yet been comprehensively assessed. Given numerous studies in rodents on steroids, predominantly estradiol, and its association with cerebral vasospasm after experimental SAH, the idea that steroids might be involved in processes linked to cerebral vasospasm or functional outcome remains to be investigated in more detail. This investigation analyzed the correlations between transcranial Doppler flow velocities in cerebral arteries and concentrations of estradiol, progesterone and testosterone in serum and CSF of patients with aSAH. This data was collected during the first two weeks after the bleeding as this period generally comprises the maximal narrowing of the cerebral arteries [1, 2]. The results showed very weak correlations between the transcranial Doppler flow velocities and the steroid concentrations in both compartments.

The neuroprotective influences attributed to estradiol in aSAH comprise vasodilatory effects and general neuroprotective mechanisms. With regard to the vasodilatory effects of estradiol, studies focused on nitrogen monoxide (NO), reactive oxygen species (ROS) and endothelins (ET) since endothelial damage and inflammation after aSAH remain discussed as key determinants of cerebral vasospasm. Various nitric oxide synthase (NOS) enzymes are identified, there among endothelial NOS (eNOS), neuronal NOS (nNOS) and inducible NOS (iNOS) [8]. In ovine endothelial cells, Chen et al. demonstrated vasodilatory effects mediated by estradiol’s capacity to activate eNOS [9]. Likewise, an in vitro study by Nevzati et al. in human umbilical and brain endothelial cells treated with estradiol a rise in NO and eNOS concentrations was demonstrated [10]. Also, Selles et al. showed in endothelial cells of rat aorta that treatment with estradiol and progesterone enhanced NOS activity [11]. In a canine SAH model, Khurana et al. found a protective effect of recombinant eNOS against cerebral vasospasm [12]. Studies have also addressed the issue of inflammation as a critical factor for vasospasm and evaluated the influences of steroids. SAH may entail formation of oxy-hemoglobin and inflammatory cascades with an increase of ROS thereby decreasing NO levels and thus facilitating cerebral vasoconstriction [3, 13, 14]. Ding et al. further discussed that vascular inflammation may enhance iNOS activity resulting in an increased ROS generation hence promoting inflammatory pathways and cerebral vasospasm [2]. In this respect Handa et al. found in primates good correlations between angiographic vasospasm and inflammation in cerebral vessels [3]. In a rodent model of SAH Lin et al. assessed the extent of vasospasm by measuring cross-section surfaces of the basilar artery and analyzing the expressions of eNOS and iNOS after treatment with estradiol [15]. In the view of Lin et al., the findings indicate vasodilatory effects of estradiol by averting an increase of iNOS expression and maintaining regular eNOS levels [15]. Zancan et al. demonstrated in smooth muscle cell cultures of rat aorta that estradiol decreased the quantity and activity of iNOS presumably via the estrogen receptor [16]. Likewise, in a rat model of SAH, Shih et al. showed that treatment with estradiol inhibited vasospasm as well as elevations of iNOS protein levels [17]. In the context of endothelial damage other studies focused on endothelins (ET) and its vasoconstrictive properties [18, 19]. As ET-1 may foster cerebral vasoconstriction in aSAH, Lin et al. investigated the effect of treatment with estradiol on ET-1 levels and cerebral vasospasm in a SAH rat model [20]. Lin et al. found a significant correlation between cross-section surfaces of the basilar artery and concentrations of ET-1 with a decrease of ET-1 levels in the rodents treated with estradiol thus suggesting an advantageous impact of estradiol on vasospasm [20]. Interestingly, Macia et al. found increased ET levels in patients with poor neurological condition apparently independent of cerebral vasospasm [21].

The neuroprotective influences of progesterone and testosterone have been investigated to a lesser extent. In a rat model with induced SAH Chang et al. showed that both the vasospasm and the lowering of eNOS protein levels in these rodents could be counteracted by progesterone [5]. In a mice SAH model Turan et al. demonstrated that progesterone treatment decreased cerebral vasospasm and had beneficial effects on behavior patterns [22]. However, as mentioned above, Zancan et al. demonstrated in cells cultures of rat aorta that estradiol decreased the content of iNOS but such an effect could not be shown with progesterone [16]. The neuroprotective relevance of testosterone in SAH remains rather undetermined as studies are scarce. In rabbits with induced SAH, Gurer et al. reasoned that intraperitoneal application of testosterone protected against cerebral vasospasm and neuronal damage [23]. Selles et al., however, noted that the enhanced NOS activity in endothelial cells of rat aorta after treatment with estradiol and progesterone could not be shown for testosterone [11].

In the present study, in humans with aSAH, merely very weak correlations were found between transcranial Doppler flow velocities in cerebral arteries and concentrations of estradiol, progesterone and testosterone in serum or CSF. These results suggest that the influence of the respective steroids in serum or CSF on cerebral vascular tone is limited. In contrast, the aforementioned studies in rodents with experimental SAH demonstrated beneficial effects of estradiol and progesterone on cerebral vasospasm, often using cross-section surfaces of cerebral arteries as a parameter [5, 15, 17, 20]. In the view of these studies, the findings in rodents appear not to be in accordance with the results of our study in humans. However, the comparability of studies using different parameters to evaluate cerebral vasospasm, such as cross-section of arteries and Doppler flow velocities respectively, may be limited. To the best of our knowledge, there are no studies in rodents on flow velocities in cerebral arteries after experimental SAH. In addition, caution is required when comparing the findings in rodents with the findings in humans. Lin et al. also stated in their study in rodents on the attenuation of cerebral vasospasm after experimental SAH by estradiol that their findings in rats may not necessarily be transferred to humans [4]. These considerations apply and complicate the interpretation of the results in the present study.

In that regard, the discussion of the results of the present study should include thoughts on concepts of steroid metabolism. The steroid hormones progesterone, testosterone and estradiol are produced in peripheral steroidogenic tissues pursuing effects in the periphery but may cross the blood–brain barrier and act in the central nervous system [24, 25]. Within the brain, steroids can also be synthesized de novo [26–28] although these processes have not yet been fully understood [25, 27, 29–31]. Additionally, steroids may be converted by neural cells into other metabolic products [32, 33]. These factors contribute to the complexity of the processes in which steroids and its metabolites may influence the functioning of the nervous system and related structures as for example blood vessels. Further research is needed to clarify these aspects. In our previous study on males without neurological disorders or diseases only weak to very weak correlations were found for estradiol, progesterone and testosterone between the CSF and serum compartments thus suggesting that concentrations in the periphery do not reflect concentrations in the central compartments [34].

The influence of the patient’s sex on cerebral vasospasm has long been a matter of debate. In a large retrospective study, Darkwah Oppong et al. addressed the role of age and sex in symptomatic cerebral vasospasm and found females aged between 55 and 74 years to be more at risk of developing symptomatic cerebral vasospasm in comparison to males of the same age [35]. Darkwah Oppong et al. suggested that the elevated risk could be in part attributed to the loss of estrogen after menopause. In contrast, Germans et al. analyzed several studies using a logistic regression model and showed that female sex is associated with an elevated risk of delayed cerebral ischemia but was not related to menopause [36]. In the present study, the gender specific correlation analysis revealed no further aspects with regard to the overall cohort as the correlation coefficients between transcranial Doppler flow velocities and steroid concentrations remained weak for males and very weak for females. The assessment of whether females are more likely to develop cerebral vasospasm after aSAH remains controversial [36–38] and needs further investigation.

It is understood that a poor neurological condition or a neurological deterioration may be due to cerebral vasospasm but a poor neurological condition can also be attributed to proapoptotic mechanisms unrelated to cerebral vasospasm [2, 39]. Studies in rodents suggest that steroids may have favorable influences on apoptotic pathways, there among studies in rodents with traumatic and ischemic brain injury [40–43] as well as studies in rodents with experimental SAH [4, 44–47]. However, in the present study, with regard to possible general neuroprotective effects of steroids, the group comparisons between steroid concentrations in the respective compartments and the grading of the Hunt & Hess scale, the grading of the Glasgow Outcome scale and the presence of cerebral infarction revealed no significant differences. Interestingly, in our previous research evaluating cognitive performance and histological damage in rats with experimental SAH no differences were found between rodents with different hormonal status [48].

The strength of the present study is its clinical approach with a study cohort consisting of humans with aSAH as rodent models of experimental SAH may not reliably represent the pathophysiological processes of spontaneous aSAH. To the best of our knowledge, there are no investigations in humans with aSAH on correlations between transcranial Doppler flow velocities in cerebral arteries and concentrations of steroids in blood or CSF. In the present study, analyses of estradiol, progesterone and testosterone were performed in both CSF and blood thus not relying solely on measurements in the blood compartment which may not adequately describe concentrations in the CSF [34]. A further strength lies in the multiple measurement points after the initial bleeding resulting in approximately 280 observations for the calculation of each correlation.

Limitations of the present study must be taken into account. Measurements of steroid levels have long been discussed and several methods are described in the literature [49], e.g. liquid chromatography, radioimmunoassay, enzyme immunoassay, competitive protein binding assay, gas chromatography-mass spectrometry. The electro-chemiluminescence immunoassay utilized in this investigation was also applied by Schonknecht et al. for the determination of estradiol concentrations in CSF of humans affected by Alzheimer`s disease [50] as well as in our previous study on males without neurological disorders for the measurement of estradiol, progesterone and testosterone in CSF and serum [34]. Steroid concentrations in CSF were also determined with an enzyme immunoassay by Kawass et al. and Brundu et al. [51, 52]. Still, method-based uncertainties cannot be precluded. Another limitation is that steroid levels representing the central compartment were determined in CSF; these measurements are associated with the incertitude as to whether they appropriately parallel brain parenchyma levels. Consequently, the findings of this study regarding correlations between flow velocities in cerebral arteries and central steroid concentrations are based on levels in CSF but not on brain parenchyma levels.

## Conclusions

In humans with aSAH, the present study demonstrated very weak correlations between transcranial Doppler flow velocities in cerebral arteries and concentrations of estradiol, progesterone and testosterone in CSF and serum. These results suggest a limited influence of the respective steroids on cerebral vascular tone although vasodilatory effects of steroids on cerebral vasospasm have been indicated in rodent studies. Cerebral vasospasm may entail neurological deficits but neurological deterioration in the absence of vasospasm is also recognized, and pathways remain complex and possibly interrelated. Thus, the implication of steroids in these processes needs further investigation.

## Data Availability

The datasets used and/or analyzed during the current study are available from the corresponding author on reasonable request.

## Abbreviations

aSAH: Aneurysmal subarachnoid hemorrhage
CSF: Cerebrospinal fluid
SAH: Subarachnoid hemorrhage
TCD: Transcranial Doppler sonography
